# Genome-wide association analysis of anti-TNF-α treatment response in Chinese patients with psoriasis

**DOI:** 10.3389/fphar.2022.968935

**Published:** 2022-08-19

**Authors:** Yunqing Ren, Ling Wang, Huatuo Dai, Guiying Qiu, Jipeng Liu, Dianhe Yu, Jianjun Liu, Cheng-Zhi Lyu, Lunfei Liu, Min Zheng

**Affiliations:** ^1^ Department of Dermatology, The Children’s Hospital, Zhejiang University School of Medicine, National Clinical Research Center for Child Health, Hangzhou, Zhejiang, China; ^2^ Department of Dermatology, The Second Affiliated Hospital, Zhejiang University School of Medicine, Hangzhou, Zhejiang, China; ^3^ Genome Institute of Singapore, Agency for Science, Technology and Research, Singapore, Singapore; ^4^ Department of Medicine, Yong Loo Lin School of Medicine, National University of Singapore, Singapore, Singapore; ^5^ Department of Dermatology, Dalian Dermatosis Hospital, Dalian, China; ^6^ Department of Dermatology, The Fourth Affiliated Hospital, Zhejiang University School of Medicine, Yiwu, Zhejiang, China

**Keywords:** psoriasis, genome-wide association analysis (GWAS), anti-TNF-α agents, treatment response, SNP

## Abstract

**Background:** TNF-α inhibitors are effective biological agents for treating psoriasis, but the treatment responses differ across patients. This study aimed to identify genetic biomarkers of anti-TNF-α response in Chinese psoriasis patients using a genome-wide association approach.

**Methods:** We recruited two independent cohorts of Chinese psoriasis patients administered etanercept biosimilar (with or without methotrexate). We identified 61 and 87 good responders (PASI improvement ≥75%), 19 and 10 poor responders (PASI improvement <50%) after 24 weeks treatment in the two cohorts, respectively. Then we performed genome-wide association studies (GWAS) on anti-TNF-α response in each cohort independently, followed by a fixed-effects inverse-variance meta-analysis in the 148 good and 29 poor responders.

**Results:** We tested genetic associations with >3 million genetic variants in either cohort. Meta-analysis identified significant associations within seven loci at *p* < 10^−5^, which also showed consistent association evidence in the two cohorts. These seven loci include rs2431355 (OR = 6.65, *p* = 4.46 × 10^−7^, *IQGAP2-F2RL2* on 5q13.3), rs11801616 (OR = 0.11, *p* = 1.75 × 10^−6^, *SDC3* on 1p35.2), rs3754679 (OR = 0.17, *p* = 7.71 × 10^−6^, *CNOT11* on 2q11.2), rs13166823 (OR = 0.09, *p* = 3.71 × 10^−6^, *IRF1-AS1* on 5q31.1), rs10220768 (OR = 5.49, *p* = 1.48 × 10^−6^, *NPAP1* on 15q11.2), rs4796752 (OR = 5.56, *p* = 1.49 × 10^−6^, *KRT31* on 17q21.2), and rs13045590 (OR = 0.08, *p* = 9.67 × 10^−7^, *CTSZ* on 20q13.3). Of the seven SNPs, six SNPs showed significant eQTL effect (*p* < 1 × 10^−6^) for several genes in multiple tissues.

**Conclusion:** These results suggest novel biological mechanisms and potential biomarkers for the response to anti-TNF therapies. These findings warrant further validation.

## Introduction

Psoriasis is an immune-mediated, chronic, inflammatory skin disorder influenced by genetic and environmental factors (Boehncke and Schon 2015; Greb et al., 2016). It is characterized by infiltration of leucocytes into the dermis and *epidermis*, excessive proliferation and changes in keratinocytes differentiation, leading to red, scaly skin, abnormal desquamation and defects in epidermal barrier. The incurable disease and the need for long-term medication bring a heavy life and psychological burden on patients. In the past decade, several biological agents have been exploited for the treatment of moderate to severe psoriasis greatly improved the situation of the patients. Among them, TNF-α inhibitors such as etanercept, adalimumab and infliximab are the most commonly used biologic drugs. However, there is considerable heterogeneity in the response to this drugs whose efficacy is range from 50% to 80% (Boehncke and Schon 2015). Accompanied with the expensive treatment expense and the potential side effects associated with the treatment, therefore, screening patients who are suitable for TNF-α inhibitors, in other words, patients with better efficacy of treatment may have a certain clinical significance.

Previous studies have identified several clinical factors such as dermoscopic hemorrhagic dots and BMI at baseline that influence the response to anti-TNF-α therapy in psoriasis (Lallas et al., 2016; Zweegers et al., 2017). Consequently, effort has been also put into the identification of genetic variation predicting anti-TNF treatment outcome. Most of these pharmacogenetics studies are candidate gene based, focusing on polymorphisms in genes known to be involved in psoriasis susceptibility or immune system such as TNF-α signaling pathways, including *TNF*, *TNF* receptor superfamily member 1A (TNF*RSF1A*), *TNFRSF1B*, *TNF-α*-induced protein 3 (*TNFAIP3*), *IL12B/IL23R*, *CARD14*, *LCE*, *HLA-Cw6* and so on (Vasilopoulos et al., 2012; Gallo et al., 2013; Batalla et al., 2015; Coto-Segura et al., 2016; Liu et al., 2016; Ovejero-Benito et al., 2017; Dand et al., 2019; van den Reek et al., 2017; Song et al., 2015). However, the majority of these studies have generated inconsistent results with small sample size and without independent validation.

GWAS has been recently used to identify new genetic biomarkers associated with the differential response to anti-TNF agents (Liu et al., 2008; Umicevic Mirkov et al., 2013; Honne et al., 2016; Julia et al., 2016), and only two studies were performed in psoriasis. The first study included 65 psoriasis patients from Japan (Nishikawa et al., 2016), and the other one included 243 psoriasis patients from Spain (Ovejero-Benito et al., 2020). However, no genome wide significant SNPs have been identified. The majority of the SNPs reported by GWAS are not known psoriasis susceptibility genes. In addition, none of the SNPs reported by candidate gene studies were confirmed by the GWAS analyses.

Here, we report a meta-GWAS analysis of two independent cohorts with a total of 212 patients treated with etanercept biosimilar. We have identified seven associated genetic biomarkers of anti-TNF drug response in Chinese Han population, which provided novel biological insight for clinical response of TNF-α inhibitor treatment.

## Materials and methods

### Patient cohorts and clinical evaluation

Two cohorts of psoriasis patients were included in this study. Cohort 1 included 103 patients and cohort 2 comprised 109 patients. All the patients 1) were Chinese descent; 2) had a psoriasis area and severity index (PASI) score >10 and an affected body surface area (BSA) > 10% at baseline; 3) were treated with etanercept biosimilar 50 mg per week. While the patients of the cohort 1 were treated only with etanercept biosimilar, the cohort 2 was from a clinic trial of combination of etanercept with methotrexate, where the patients were treated with etanercept biosimilar 50 mg per week and a stable dose of methotrexate 15 mg per week (definite dose depends on the patient’s tolerance). Methotrexate is one of the most common and conventional disease-modifying anti-rheumatic drugs used for psoriasis and psoriatic arthritis. Detailed clinical parameters collected includes present age, sex, disease duration, smoking and alcohol drinking history, family history, psoriatic arthritis, previous systemic therapies, and PASI score at first screening and during subsequent visits. The study was approved by the local institutional ethics review board, the second affiliated hospital of Zhejiang University school of medicine, and written informed consent was obtained from all patients.

### Efficacy measurements

We used PASI score to evaluate the clinical severity of disease for all the patients. We calculated response as the percentage improvement in the PASI score at 12 and 24 weeks compared with that at baseline. In this study, the patients had a better therapeutic effect at week 24 than that at week 12. Patients were categorized as poor responders, if their PASI improvement rates were <50%, or good responders, if their PASI improvement rates were ≥75%. Similar to previous studies, moderate responders (whose PASI improvement rates were between 50% and 75%) were excluded for association analysis (Masouri et al., 2016; Mendrinou et al., 2016).

### Genotyping and quality control

Genomic DNA of each sample was extracted from peripheral blood using standard protocols. Genotyping analysis of the two cohorts was conducted using the Illumina Infinium Asian Screening Array-24 v1.0 BeadChip. We performed quality control (QC) on the two cohorts respectively. We excluded SNPs from X, Y, and mitochondrial chromosomes from GWAS analysis. All samples had good quality with high call rate (>95%) and no extreme heterozygosity (<3SD). Cryptic relatedness between individuals was identified through identity by descent analysis using PLINK (see URL) and two first degree relative pairs were identified in cohort 2. One sample of each pair with lower SNP call rate were removed. Principle component analysis was performed using PLINK, one outlier was identified and removed in cohort 1. Totally, 102 samples for cohort 1 and 107 samples for cohort 2 passed QC and remained in further analysis.

### Genotype imputation and quality control

To increase overall coverage of the genome, we performed imputation using IMPUTE v2 based on the 1,000 Genomes Project reference panel from all populations (dated October 2014). We imputed on basis of samples with high call rate and the genotyped SNPs that passed quality control (call rate≥95%, HWE *p* ≥ 1 × 10^−6^, MAF≥0.01). SNP quality control after imputation were as follows: imputation quality score ≥0.8, HWE *p* ≥ 1 × 10^−6^, MAF≥10% in both good responders and poor responders.

### Statistical analyses

The difference of clinical characteristics of the patients between the two cohorts was calculated by Chisq test or *t* test using R 4.0.2. The genome-wide case-control association analysis was conducted using SNPTEST v2 (see URLs) and imputed dosage data in each cohort. The association test was performed by assuming an additive model for allelic risk, with the age, gender, BMI at baseline and the first three principal components included as covariates to adjust for study effect and population stratification. To combine the association statistics from the two independent GWASs, we performed a fixed-effects inverse-variance meta-analysis of 3478293 overlapped SNPs using META (see URLs). Cochran’s Q test and I^2^ were used to assess between-study heterogeneity.

Power calculation was performed for the top SNPs detected in the current study using Quanto (see URL) under following assumption: disease prevalence of 0.01% and a log-additive risk model. For the calculation, we used effect size estimates observed in the combined population and the observed allele frequencies in all samples. The current sample size has a moderate power (ranging from 45% to 93%) to detect the effect at a *p*-value of 1 × 10^−5^.

We first determined the SNPs and indels that are in high LD (r2 ≥0.8) with seven lead SNPs using the LD information provided by HaploReg V3 on the 1,000 Genomes phase 1 Asian (ASN) population, which identified 86 SNPs and small indels. Then the mRNA expression level (eQTL) effect was evaluated through Genotype-Tissue Expression (GTEx) portal (see URL).

## Results

### Study subjects

The baseline characteristics of the two cohorts were summarized in Table 1. For the Cohort 1, the average age of the patients at baseline was 42.5 years, with 72.6% being male. The mean PASI score at baseline was 24.4 ± 12.3, which gradually decreased during the treatment and achieved the best curative effect at 24 weeks. For the Cohort 2, the baseline average age, proportion of male and PASI score was 41.8 ± 13.2, 78.5% and 22.3 ± 9.3, respectively. There was no significant difference in baseline age, gender ratio, BMI, personal history between the two cohorts as well as the PASI score at baseline. However, the overall response to treatment in term of PASI reduction was better for the Cohort 2 than the Cohort one due to the combined using of methotrexate.

**TABLE 1:** Basic clinical characteristics of the patients in this study.

Characteristics	GWAS cohort1 (*n* = 102)	GWAS cohort2 (*n* = 107)	*p* Value
Age, years, mean ± s.d	42.5 ± 12.3	41.8 ± 13.2	6.93E-01
Gender, man, n (%)	74 (72.6%)	84 (78.5%)	4.00E-01
Psoriasis duration, years, mean ± s.d	15.5 ± 8.0	14.3 ± 10.5	3.76E-01
BMI, mean ± s.d	24.2 ± 3.1	23.9 ± 3.4	4.26E-01
Ever smoker, yes, n (%)	59 (57.8%)	53 (49.5%)	4.57E-01
Ever drinker, yes, n (%)	67 (65.7%)	81 (75.7%)	1.59E-01
Arthropathic psoriasis, n (%)	13 (12.9%)	7 (6.6%)	1.97E-01
PASI at baseline	24.4 ± 12.3	22.3 ± 9.3	1.70E-01
PASI at 12 weeks	8.3 ± 7.1	5.3 ± 5.0	4.37E-04
PASI at 24 weeks	6.1 ± 7.9	2.7 ± 4.0	1.34E-04
Good responders	61 (59.8%)	87 (81.4%)	2.61E-04
Moderate responders	22 (21.6%)	10 (9.3%)	
Non-responders	19 (18.6%)	10 (9.3%)	

Abbreviations: GWAS, Genome-Wide Association Study; BMI, body mass index; PASI, psoriasis area and severity index.

### Genome-wide association analysis

To minimize the adverse impact of the misclassification of treatment response, we just analyzed the patients of good responders (PASI improvement ≥75%) and poor responders (PASI improvement <50%) after 24 weeks treatment. There were 61 good responders and 19 poor responders for the Cohort 1, and 87 good responders and 10 poor responders for the Cohort 2. Because the number of poor responders was moderate for both cohorts (19 for the cohort 1 and 10 for the cohort 2), only the SNPs whose MAFs were above 10% in the samples of poor responders were included in the association analysis. We further performed PCA analysis of all the patients and found that good and poor responders were well matched genetically without evidence of population stratification (Supplementary Figures S1–S3).

**FIGURE 1:**
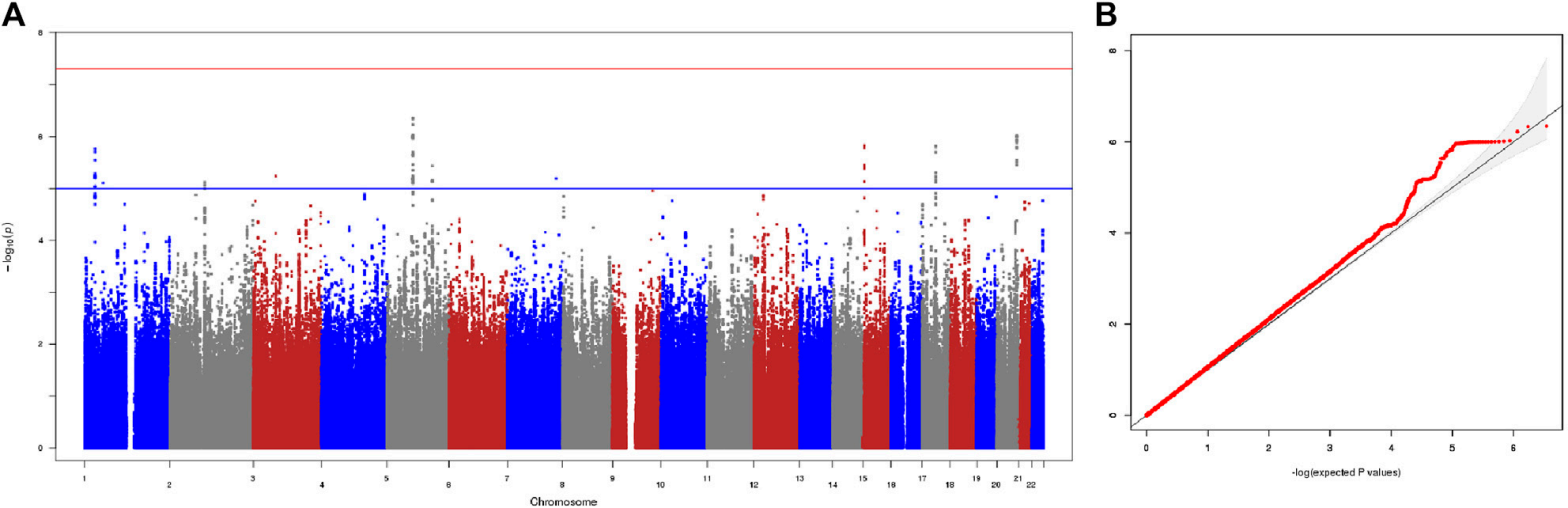
Results of meta-GWAS of the two cohorts. **(A)** The −log_10_ genome-wide *p* values using a fixed-effects inverse-variance meta-analysis calculated on the basis of data from overlapped 3478293 SNPs in the 148 good responders and 29 poor responders. The blue horizontal line presents a liberal threshold of 10^−5^ for suggestive significance. The red horizontal line indicates genome-wide significance (*p* < 5 × 10^−8^). **(B)** Quantile-quantile plots of the observed *p* values versus the expected values form *p* value of association. λgc = 1.065.

We first performed the genome-wide association analysis in the two cohorts independently: 3815035 autosomal SNPs for the Cohort 1, and 3754575 autosomal SNPs for the Cohort 2. To assess and control the genetic heterogeneity of association effect between the two independent cohorts, we performed a Meta-GWAS analysis of the two cohorts where the association results of 3478293 overlapping SNPs from the two cohorts were combined by a meta-analysis under a fixed-effects model. The λgc of Meta-GWAS analysis was 1.065, indicating a minimal inflation of the genome-wide association results due to population stratification, which is consistent with the result of PCA analysis. Consistently, the quantile-quantile plot of the observed *p* values for association (Figure 1) showed that the distribution of the logarithms of the observed *p* values largely fits the null distribution.

The Meta-GWAS analysis revealed significant association at seven loci with a *p*-value less than 10^−5^ (Table 2). The top association was observed at rs2431355 (OR = 6.65, *p* = 4.46 × 10^−7^, *IQGAP2-F2RL2* on 5q13.3), and the other significant associations were found at rs11801616 (OR = 0.11, *p* = 1.75 × 10^−6^, *SDC3* on 1p35.2), rs3754679 (OR = 0.17, *p* = 7.71 × 10^−6^, *CNOT11* on 2q11.2), rs13166823 (OR = 0.09, *p* = 3.71 × 10^−6^, *IRF1-AS1* on 5q31.1), rs10220768 (OR = 5.49, *p* = 1.48 × 10^−6^, *NPAP1* on 15q11.2), rs4796752 (OR = 5.56, *p* = 1.49 × 10^−6^, *KRT31* on 17q21.2), and rs13045590 (OR = 0.08, *p* = 9.67 × 10^−7^, *CTSZ* on 20q13.3). For all the seven loci, the minor allele of the top SNP was associated with poor response. For each of the seven loci, the association was detected in both cohorts, and the estimated genetic effect of association (OR beta value) was consistent between the two cohorts without any evidence of genetic heterogeneity (Table 2). Furthermore, for each of the seven loci, the evidence of association was supported by both genotyped and imputed SNPs. The regional plots of the seven loci are provided in Supplementary Figures S4–S10.

**TABLE 2:** Meta-analysis results of the seven loci for anti-TNF response in psoriasis.

Chr	Implicated Genes	Top SNP in Region	Position	Allele	GWAS Cohort1				GWAS Cohort2				Meta-GWAS		
					F_G	F_P	*p* Value	Or (95%CI)	F_G	F_P	*p* Value	Or (95%CI)	*p* Value	Or (95%CI)	P_heterogeneity
1	*SDC3*	rs11801616	31311684	G	0.11	0.39	1.25E-04	0.10 (0.03–0.33)	0.17	0.35	4.29E-03	0.12 (0.03–0.51)	1.75E-06	0.11 (0.04–0.27)	8.93E-01
2	*CNOT11*	rs3754679	101599393	G	0.39	0.57	6.89E-04	0.20 (0.08–0.50)	0.35	0.58	3.06E-03	0.13 (0.03–0.50)	7.71E-06	0.17 (0.08–0.37)	5.95E-01
5	*IQGAP2/F2RL2*	rs2431355	75934205	T	0.52	0.23	6.79E-05	6.97 (2.68–18.12)	0.51	0.15	1.90E-03	6.22 (1.96–19.71)	4.46E-07	6.65 (3.19–13.89)	8.81E-01
5	*IRF1-AS1*	rs13166823	131752644	G	0.14	0.31	1.30E-04	0.07 (0.02–0.28)	0.22	0.40	8.05E-03	0.13 (0.03–0.58)	3.71E-06	0.09 (0.03–0.25)	6.13E-01
15	*NPAP1*	rs10220768	24319693	C	0.65	0.32	4.41E-06	7.46 (3.16–17.54)	0.51	0.30	6.12E-02	3.08 (0.95–10.01)	1.48E-06	5.49 (2.74–10.97)	2.36E-01
17	*KRT31*	rs4796752	39629843	C	0.63	0.40	1.52E-03	4.35 (1.75–10.78)	0.59	0.25	2.04E-04	7.93 (2.66–23.67)	1.49E-06	5.56 (2.76–11.18)	4.06E-01
20	*CTSZ*	rs13045590	57575102	T	0.11	0.25	3.15E-04	0.09 (0.22–0.33)	0.18	0.37	8.73E-04	0.06 (0.01–0.32)	9.67E-07	0.08 (0.03–0.21)	7.95E-01

Abbreviations: Chr, chromosome; SNP, single nucleotide polymorphisms; F_G, frequency of good responders; F_P, frequency of poor responders; OR, odds ratio; CI, confidence interval for odds ratio.

The italic values represent gene names.

To further investigate the association of these seven SNPs at 12 weeks, a fixed-effects inverse-variance meta-analysis was also performed in the patients of good responders (PASI improvement ≥75%) and poor responders (PASI improvement <50%) after 12 weeks treatment. There were 46 good responders and 31 poor responders for the Cohort 1, and 65 good responders and 15 poor responders for the Cohort 2. These seven top SNPs association analysis results at 12 weeks are shown in Supplementary Table S1, which showed consistent trends of association as the analysis at 24 weeks.

In addition, we checked the associations for the 19 SNPs that were reported with *p* < 4 × 10^−5^ in the two published GWAS on anti-TNF-α response (Nishikawa et al., 2016; Ovejero-Benito et al., 2020); Two of these SNPs (rs77497886 and rs1487419 on CDH12) achieved nominal significance in our study (*p* = 0.031 and 0.032).

### EQTL analysis and functional annotation

To better understand the biological function, we have investigated the regulatory functions of these seven top significant SNPs by analyzing the information from the HaploReg (v3) and the GTEx database. Of the seven SNPs, four SNPs were found in the database, rs2431355, rs13045590, rs3754679 and rs4796752, and showed significant eQTL effect (*p* < 1 × 10^−6^) for several genes in multiple tissues. The top SNP rs2431355 conferred significant eQTL effect in multiple tissues for *F2RL2* gene (*p* = 3.70 × 10^−19^). rs13045590 was associated with significant eQTL effect (*p* = 9.50 × 10^−23^) on the expression level of *CTSZ* gene in muscle-skeletal and heart. rs4796752 was showed most significant eQTL effect (*p* = 1.80 × 10^−11^) with *KRT31* gene in skin, and rs3754679 was showed **s**ignificant eQTL with *CNOT11* gene in breast tissue with *p* = 1.00 × 10^−6^. rs11801616 and rs10220768 were absent in the database, but 13 SNPs in high LD (*r* ^2^ > 0.8) were also analyzed, which showed conferred eQTL effect for *SDC3* (*p* = 6.40 × 10^−9^) and *NPAP1* gene (*p* = 7.80 × 10^−6^) respectively. rs13166823 and SNPs in high LD had no eQTL results in GTEx portal. The eQTL analysis results were summarized in Supplementary Table S2.

## Discussion

In this study, we performed the first genome-wide association study to evaluate pharmacogenetic effect on the response to etanercept biosimilar in Chinese population. By investigating two independent cohorts of patients of psoriasis treated with etanercept biosimilar (with or without methotrexate), we were able to evaluate the robustness of the association by comparing the consistence of statistical evidences from independent samples using genetic heterogeneity test. By interrogating 3478293 SNPs in a total of 148 good responders and 29 poor responders, the current study is the largest effort in Asian population and has discovered seven loci that were significantly associated with treatment outcome. Although the statistical evidences of association for these seven loci did not reach genome-wide significance (p < 5E-08), the robustness of the association evidence is reflected by the consistent evidence from the two independent cohorts as well as supported by multiple SNPs within each locus. Furthermore, the associations at these seven loci were also supported by the treatment outcome data of these two cohorts at 12 W. As previous studies, we have excluded the subjects with moderate response (PASI improvement between 50% and 75%), to minimize the misclassification of treatment response and thus enhance the robustness of association results.

The strongest association with treatment response was found in the *IQGAP2*- *F2RL2* locus on 5q13.3. *IQGAP2* gene encodes the IQ motif containing GTPase activating protein 2, which regulates a wide variety of cellular signaling pathways. *IQGAP2* gene has been shown to function in thrombin-induced platelet cytoskeletal reorganization (Schmidt et al., 2003). In addition, the top SNP rs2431355 conferred significant eQTL effect in multiple tissues for F2RL2, which also plays an essential role in hemostasis and thrombosis. Variants in *IQGAP2* have previously been associated with platelet count and mean platelet volume (MPV) (Eicher et al., 2016). Interestingly, accumulating evidence has shown that platelet dysfunction plays an important role in the pathogenesis of psoriasis (Fan et al., 2020). Besides as the principal mediator of hemostasis and thrombosis, platelets are also immune cells that involve in immune and inflammatory processes (Zamora, Canto, and Vidal 2021). Platelets aggregation has been detected in patients with psoriasis. Several studies have suggested that MPV values were significantly higher in patients with psoriasis and correlated with disease severity, also decreased after effective biological therapy (Capo et al., 2014; Kilic et al., 2017; Fan et al., 2020). Moreover, the levels of platelet-lymphocyte complexes and platelet to lymphocyte ratio were also indicated to be prognostic biomarkers of response to anti-TNF-α treatment (Najar Nobari et al., 2020; Sanz-Martinez et al., 2020). Taking together, our current finding and previous evidences suggest that genetic variation influencing platelets activity could influence the resistance or efficacy to anti-TNF agents. Further studies confirming the functional implication of *IQGAP2/*F2RL2 in psoriasis and the response to anti-TNF agents are warranted.

In addition, 12 SNPs on chromosome 20q13 were found to be associated with treatment outcome. The most significant SNP rs13045590 was in an intron of the cathepsin Z (*CTSZ*) gene. Using the GTex data, we also found that rs13045590 was associated with significant eQTL effect on the expression level of *CTSZ* gene in muscle-skeletal and heart. CTSZ is a member of the cysteine cathepsins, which regulates various cellular physiological functions, including adhesion, migration, invasion, and maturation of immune cells. Recently this SNP was found to be associated in Platelet distribution width (PDW) (Vuckovic et al., 2020). It is also interesting to note that several variants within this locus (*CTSZ, TUBB1* and *PRELID3B*) had been previously reported to be associated with MPV and platelet count (Gieger et al., 2011; Qayyum et al., 2012). Therefore, the association identified in 20q13 has further implicated the link between platelets activity and anti-TNF-α treatment response in psoriasis. Validation studies will be required to explore this further.

Another suggestive locus is identified at 5q31.1, which is immediately adjacent to the locus that was previously reported to be associated with psoriasis and Crohn’s disease (Rioux et al., 2001; Chang et al., 2008). This locus contains a cluster of cytokine and immune-related genes including interferon regulatory factor 1 (*IRF1*), *IL5* and *IL13* as well as *SLC22A4* and *SLC22A5*. Rs13166823 is located within IRF1 antisense RNA 1(*IRF1-AS1*). The protein encoded by *IRF1* gene is a transcriptional regulator and tumor suppressor, playing a role in cell proliferation, apoptosis, DNA damage response as well as innate and acquired immune responses. Previous study revealed that IRF1 expression decreased in lesional psoriatic skin compared with nonlesional or normal skin. And the induction of IRF1 activity to IFN-γ was also greatly reduced in psoriatic keratinocytes vs. normal keratinocytes (Jackson et al., 1999). Recent studies indicated that IRF1 could mediate M1 polarization of macrophages which may contribute to psoriasis. However, Lin et al. found that TNF-α blockage inhibits M1 polarization through STAT1-and IRF1-independent pathways. Furthermore, rs2070729 in *IRF1* has been previously associated with platelet count (Gieger et al., 2011). These findings suggest that *IRF1* may be involved in the pathogenesis of psoriasis whose genetic variation may affect the response during psoriasis treatment.

Rs10220768 is located in Prader-Willi region non-protein coding RNA 4 (*PWRN4*). Using the published eQTL data, we have shown that rs10220768 and six linked SNP were associated with significant eQTL effect on the expression level of nuclear pore associated protein 1 (*NPAP1*) gene in thyroid. *NPAP1* gene encodes nuclear pore associated protein one which also located chromosome 15 and defects in this gene may be associated with Prader-Willi syndrome. A recent study has identified that *NPAP1* is one of the potential genes which associated with the response to immune checkpoint inhibitors therapy in patients with non-small cell lung cancer (Yu et al., 2019). Considering the high efficacy in psoriasis therapy of TNF-α inhibitors, it is likely that these SNP-associated genes could be involved in the response of immunotherapy in psoriasis.

The SNP rs4796752 on 17q21.2 is located within the region of the keratins genes clusters. Using the GTex data, eQTL analysis showed that rs4796752 was most significantly associated with *KRT31* gene in skin. KRT31 is a type I acidic keratin and played an essential role in maintaining the health state of hair follicles. Application of K31 improved the damaged hair in smoothness, diameter and mechanical strength (Basit et al., 2018). Previous evidence showed K31 peptides could contribute to alopecia areata induction by supporting T cell activation when presented by DCs to syngeneic naive T cells in mice (Erb, Freyschmidt-Paul, and Zoller 2013). More and more evidence showed that connection between psoriasis and hair follicles on shared molecular regulatory mechanism and therapeutic targets (Suzuki et al., 2022). Patients with psoriasis have an OR of 2.5 for developing alopecia areata. It has also been reported that alopecia may be related to psoriasis itself or systemic therapies used to treat it especially anti-TNF-α agents. Of relevance, treatment with anti-TNF-α inhibitors can also cause alopecia. Therefore, a potentially interesting connection exists between *KRT31* and psoriasis as well as the efficacy of TNF blockade.

Twelve SNPs in the locus on chromosome 1p35.2 had *p* value < 10^–5^, with the most significant SNP rs11801616 located in the 5 kb upstream of 5′ of RN7SK pseudogene 91 (*RN7SKP91*). Interestingly, seven SNPs in high LD with rs11801616 were associated with significant eQTL effect on the expression level of *SDC3* gene in Artery. Syndecan-3, encoded by *SDC3* gene, belongs to the family of heparan sulfate proteoglycans (HSPGs) and mainly involves in brain development and feeding behaviors. Recent studies suggested that *SDC3* has important roles in inflammation and angiogenesis (Arokiasamy et al., 2019). Increased expression of SDC3 has been shown on endothelia in rheumatoid arthritis and psoriasis arthritis synovium comparing to normal synovium, which is thought to have roles in angiogenesis, chemokine presentation and leukocytes extravasation (Patterson et al., 2008). Furthermore, we recently identified several pathways and functional processes including HSPG and VEGF production which are correlated with anti-TNF-a treatment (Liu et al., 2019). Therefore, *SDC3* has the possibility to be a crucial part in the pathogenesis of psoriasis and influence the therapeutic response in psoriasis.

Rs3754679 on 2q11.2 is located within Neuronal PAS domain protein 2 (*NPAS2*) gene. The eQTL analysis showed that rs3754679 was associated with significant eQTL effect on the expression level of *CNOT11* gene in mammary breast. CNOT11 was one of subunits of the CCR4 (carbon catabolite repressor 4)-NOT (Negative on TATA) complex, which plays a crucial role in post-transcriptional mRNA regulation in eukaryotes. Previous evidence showed CCR4-NOT complex contributes to repression of Major Histocompatibility Complex class II transcription (Rodriguez-Gil et al., 2017). Although the role of CNOT11 subunit and how it is integrated into the complex are still unknown, it was found to be significantly upregulated in psoriatic skin lesions. These findings suggested a potential association between CCR4-NOT and psoriasis as well as the efficacy of TNF blockade.

A major limitation of the current study is that the seven loci did not reach genome-wide significance, the same as the two previous GWAS study carried out in 65 Japanese patients and 243 Caucasian patients. Although we reduced the adverse impact of misclassification on the results by excluding the partial responders in the association analysis, the reduced sample size would decrease the statistical power of this study and thus weaken the statistical significance of the results. In addition, the treatment regime was not identical between the two cohorts. The patients of the Cohort 1 were treated with etanercept biosimilar alone, while the patients of the Cohort 2 were treated with the combination therapy of etanercept biosimilar and methotrexate. Methotrexate, a folic acid antagonist, is a conventional systemic agent used widely for psoriasis. Adding methotrexate to etanercept has been proved to increase the efficacy compared with etanercept alone (Gottlieb et al., 2012). Although the meta-analysis could control the genetic heterogeneity of association effect, it is still difficult to avoid the effect of increased efficacy on results of Cohort 2 due to combined using of methotrexate. Therefore, our current findings require further validation in independent samples and/or at a functional level.

In the present study, we have identified seven genetic loci that show evidence of influencing anti-TNF treatment response based on a meta-GWAS in 177 Chinese psoriasis patients, which might serve as predictors for treatment and guide personalized therapeutic decisions. These genetic associations also provided biological insight for how TNF-α inhibitor treatment, suggesting the existence of different biological mechanisms through which TNF-α inhibitors exert their immunomodulatory role in psoriasis.

## URLs:

PLINK: http://www.cog-genomics.org/plink/1.9/

IMPUTE: https://mathgen.stats.ox.ac.uk/impute/impute_v2.html

SNPTEST: https://mathgen.stats.ox.ac.uk/genetics_software/snptest/snptest.html#download

META: http://mathgen.stats.ox.ac.uk/genetics_software/meta/meta.html#

Quanto: https://bio.tools/QUANTO

GTEx: https://gtexportal.org/home/

## Data Availability

The datasets presented in this study can be found in online repositories. The names of the repository/repositories and accession number(s) can be found below: https://ngdc.cncb.ac.cn/gvm/getProjectDetail?project=GVP000002, GVP000002.
