# Supplementary figures and images for: Genome-wide association analysis of anti-TNF-α treatment response in Chinese patients with psoriasis
Source: Front Pharmacol. 2022 Aug 19;13:968935. doi: 10.3389/fphar.2022.968935 (PMC9437453; doi:10.3389/fphar.2022.968935)

Plotted SNPs

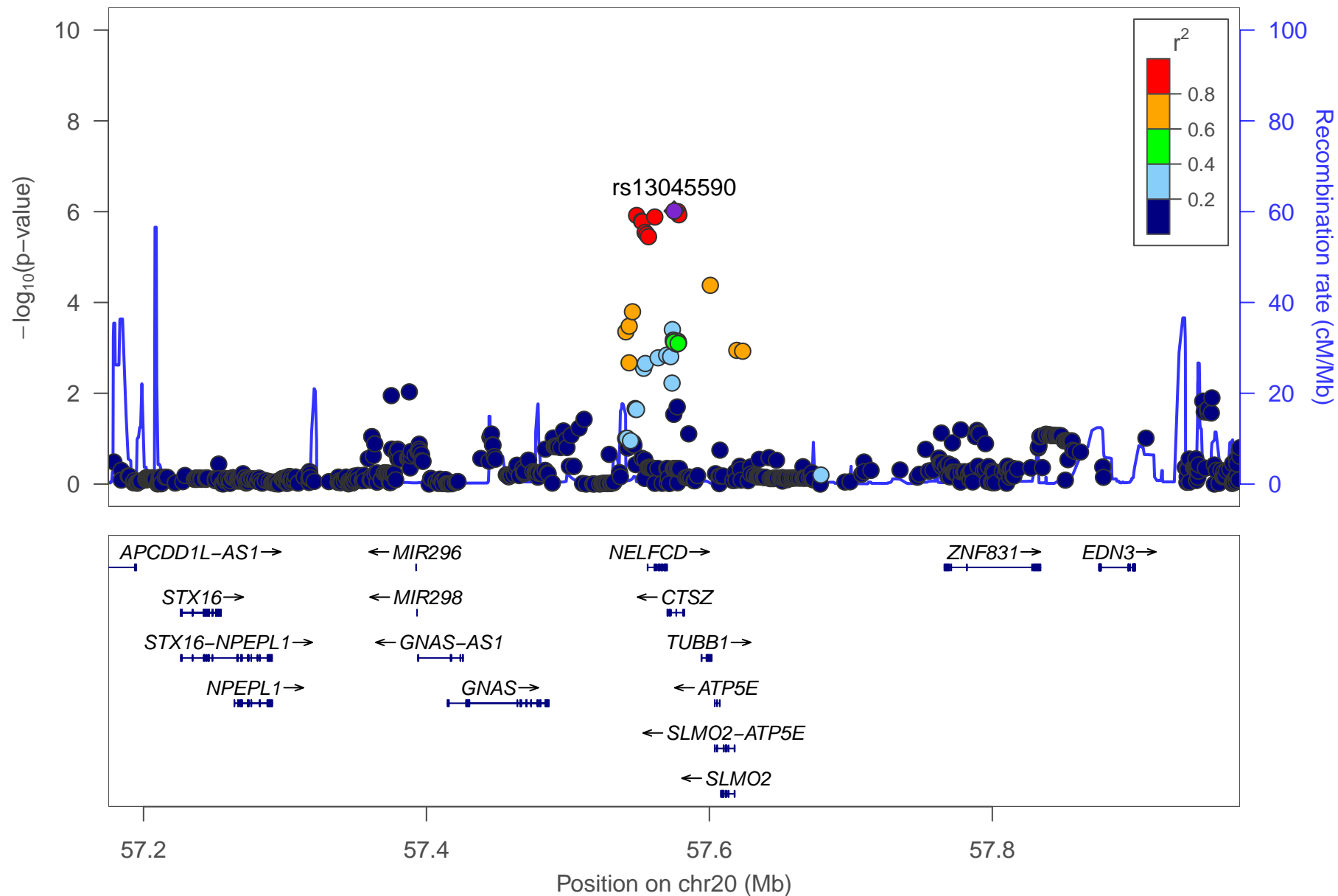

Supplement: Supplementary file 1 [file DataSheet7.PDF]

Plotted SNPs

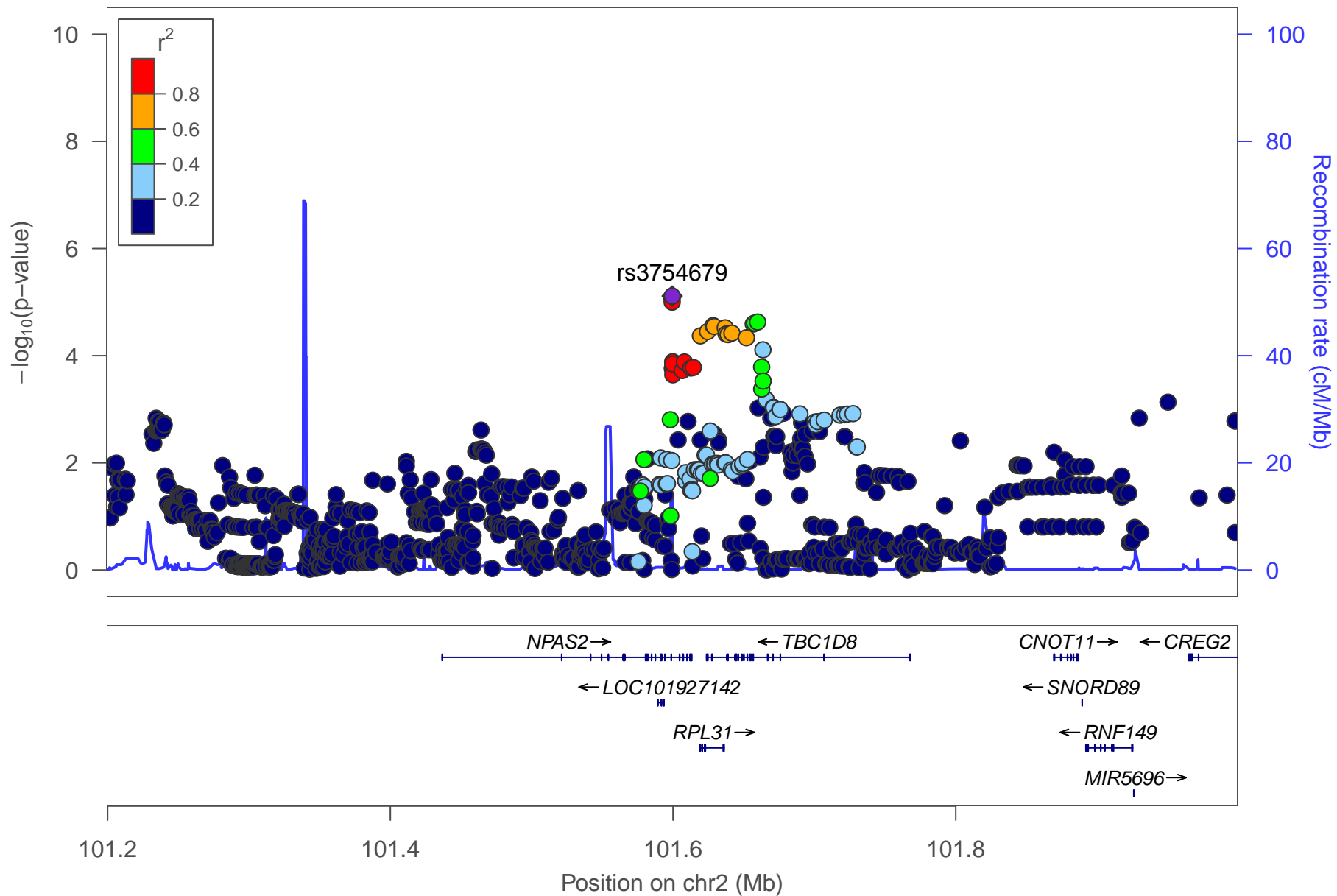

Supplement: Supplementary file 2 [file DataSheet2.PDF]

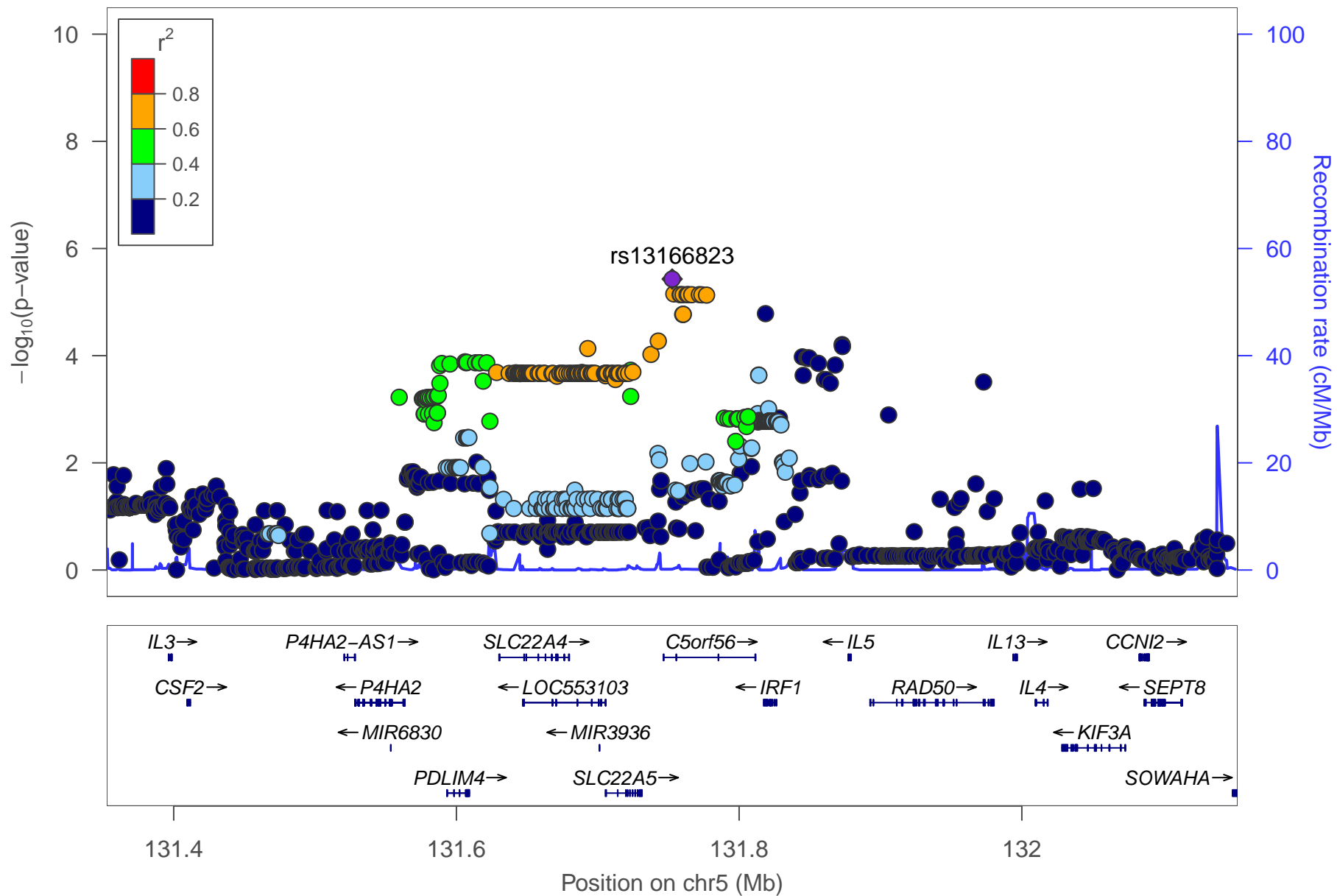

Supplement: Supplementary file 3 [file DataSheet4.PDF]

Plotted SNPs

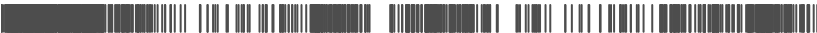
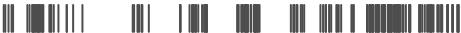
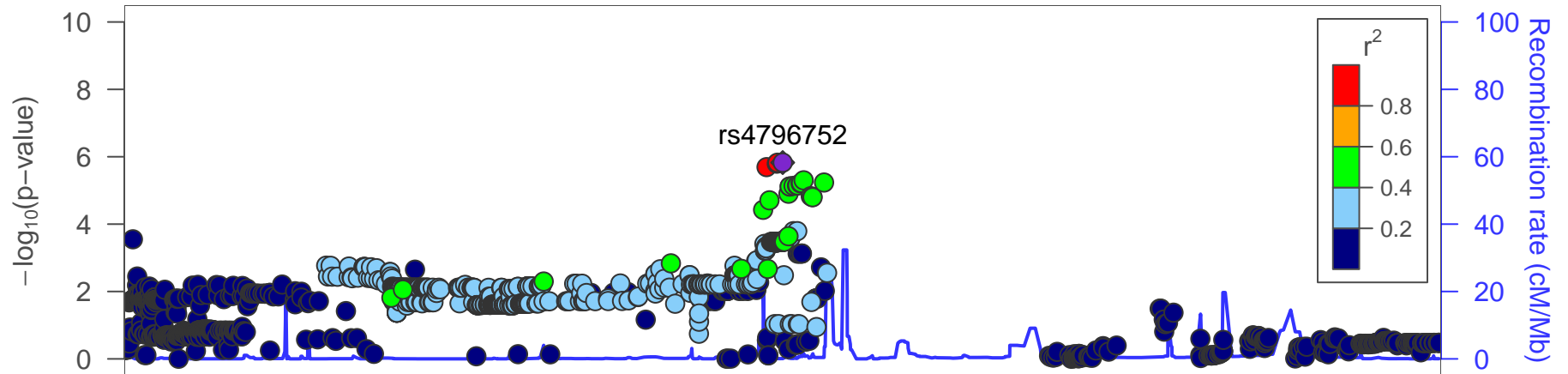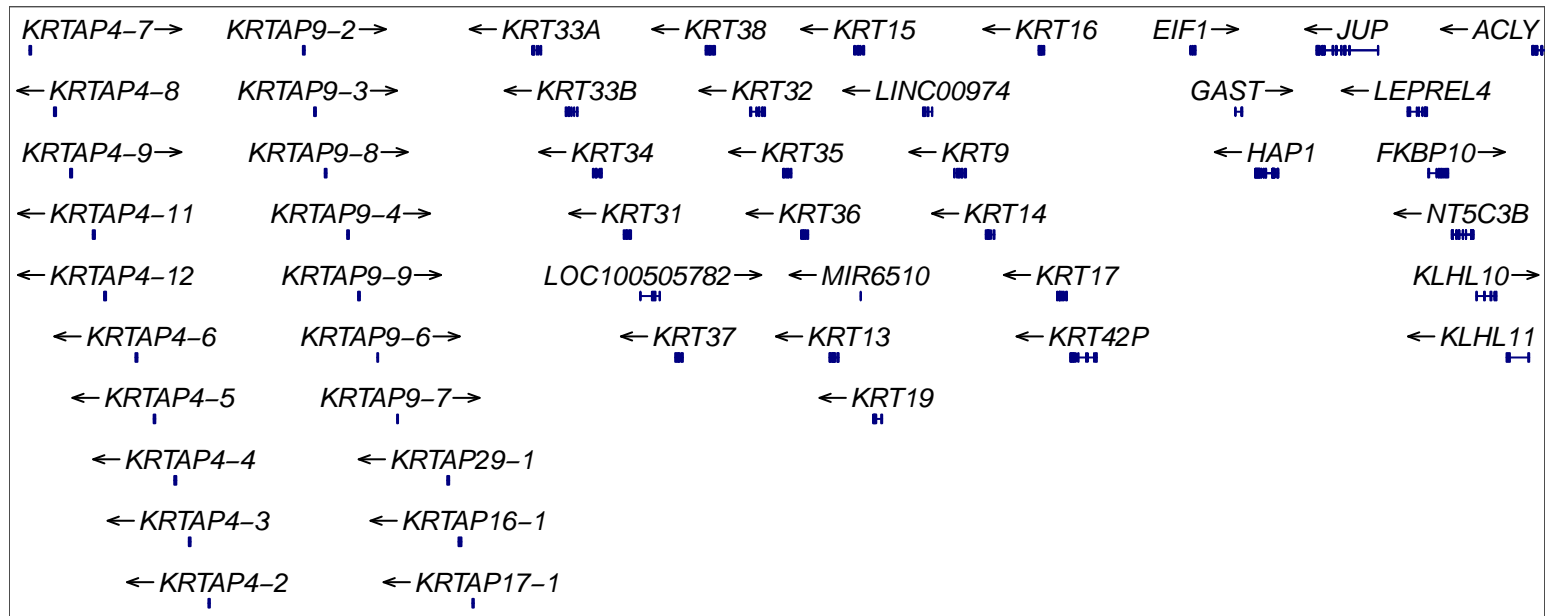

2 genes  
omitted

39.4

39.6

39.8

40

Position on chr17 (Mb)

Supplement: Supplementary file 4 [file DataSheet6.PDF]

Plotted SNPs

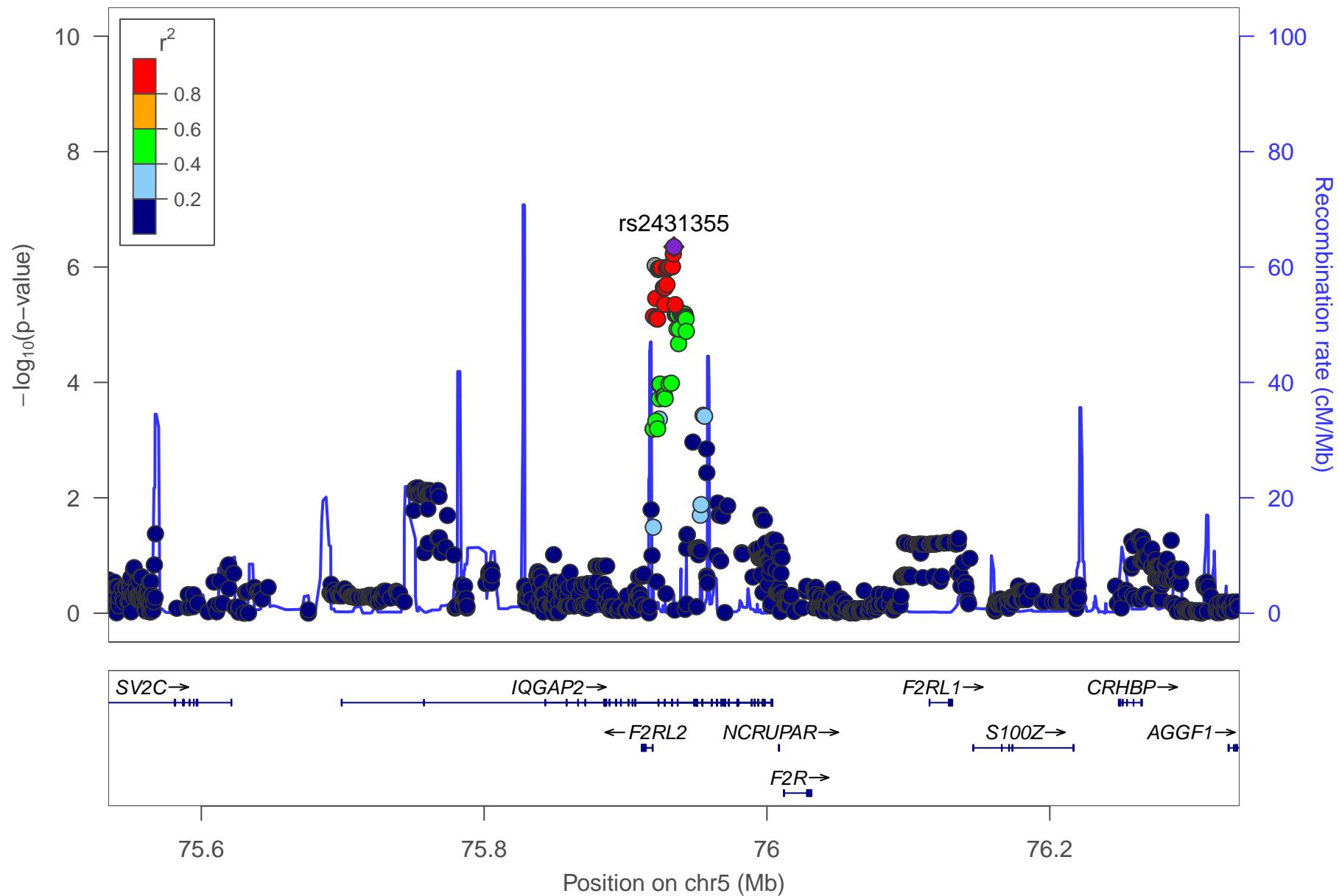

Supplement: Supplementary file 6 [file DataSheet3.PDF]

Plotted SNPs

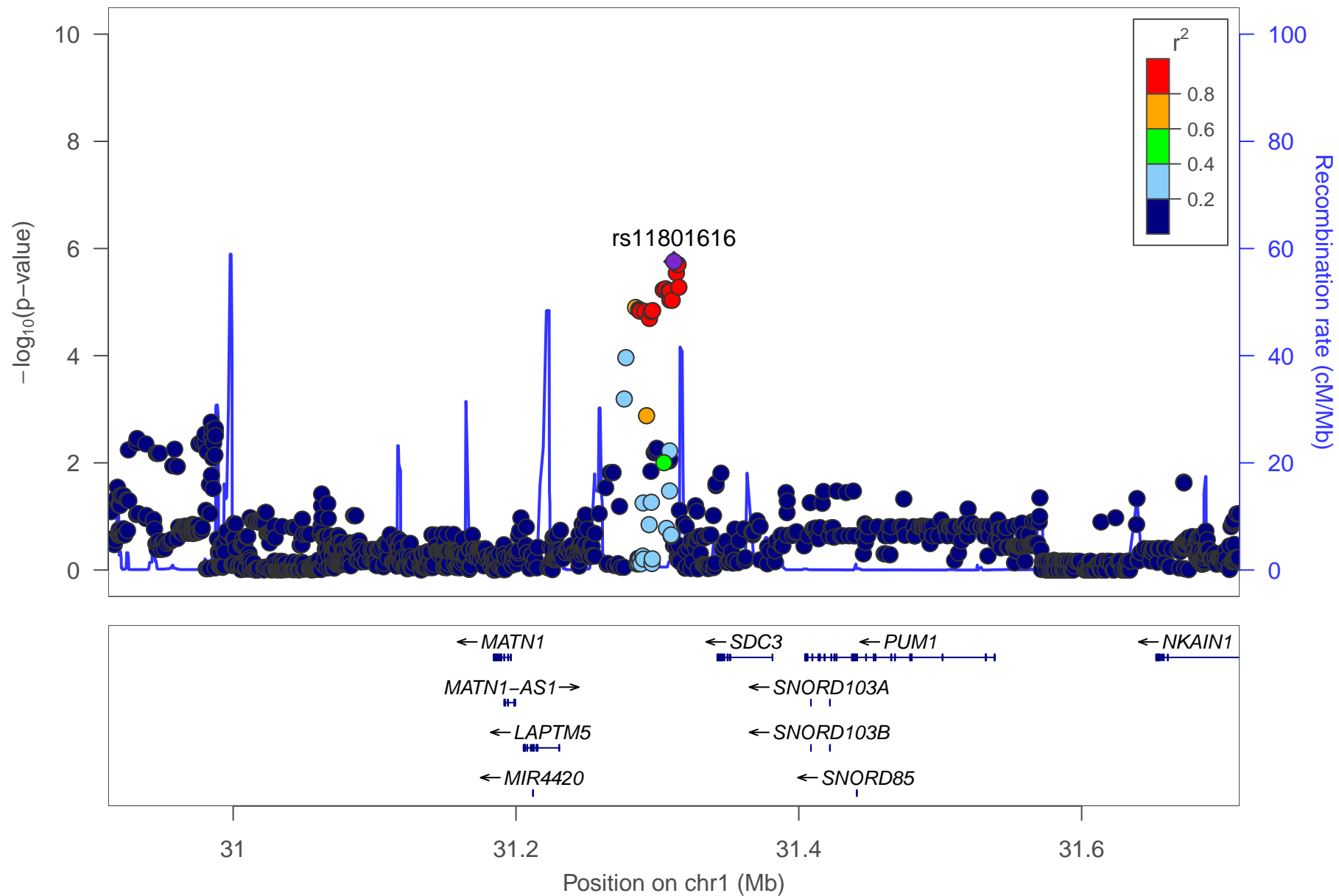

Supplement: Supplementary file 7 [file DataSheet1.PDF]

Plotted SNPs

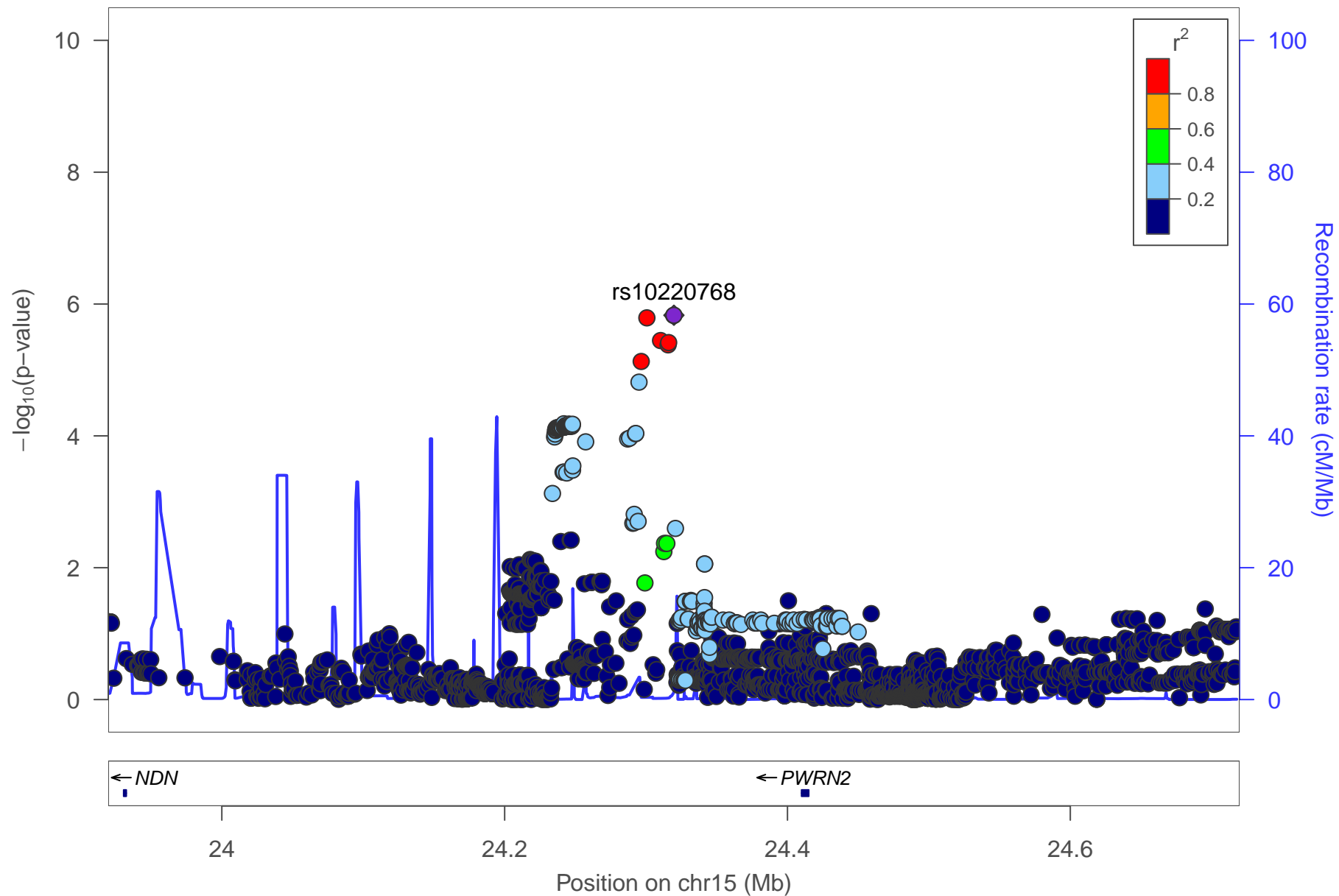

Supplement: Supplementary file 8 [file DataSheet5.PDF]

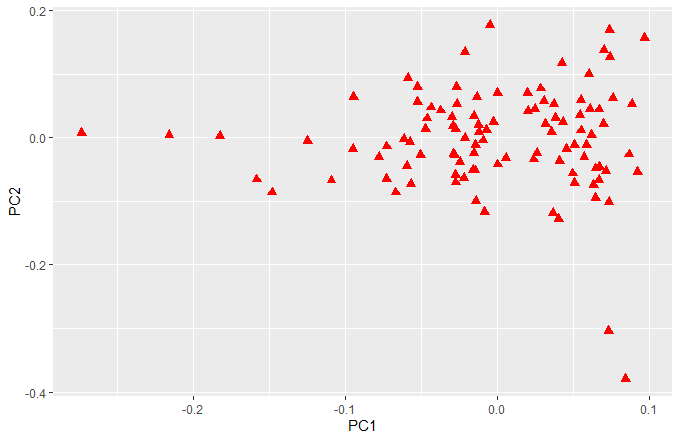

Supplement: Supplementary file 9 [file Image2.PNG]

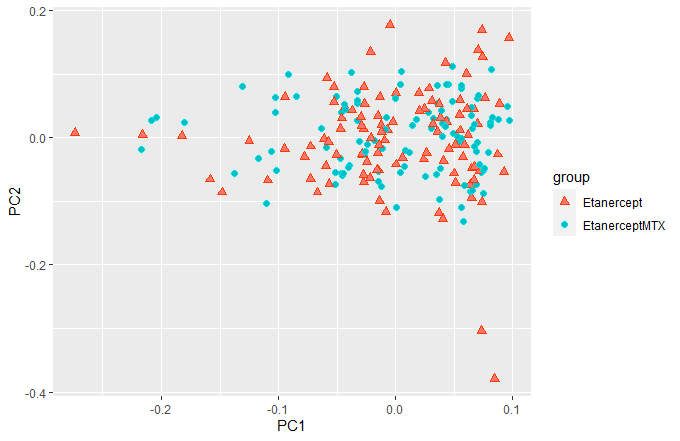

Supplement: Supplementary file 10 [file Image1.PNG]

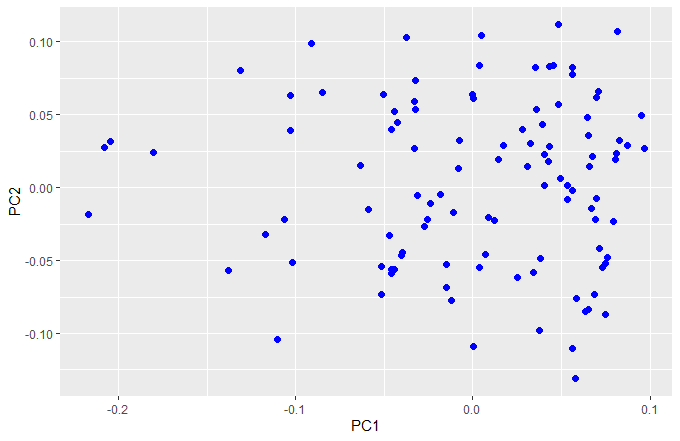

Supplement: Supplementary file 11 [file Image3.PNG]
