# Supplementary material for: Genome-wide association analysis of anti-TNF-α treatment response in Chinese patients with psoriasis
Source: Front Pharmacol. 2022 Aug 19;13:968935. doi: 10.3389/fphar.2022.968935 (PMC9437453; doi:10.3389/fphar.2022.968935)
Supplement: Supplementary file 5 [file Table1.DOCX]

**Genome-wide association analysis of** **anti-TNF-α treatment response in Chinese patients with psoriasis**

**Supplementary Figure legends**

Plots of principal components from the principal components analysis using 209 participant samples. The orange points are cohort 1 and the light blue points are cohort 2.

Figure S1: PCA plot of the first and second components of 209 participant samples.

Figure S2: PCA plot of the first and second components of Cohort 1.

Figure S3: PCA plot of the first and second components of Cohort 2.

Association results for seven loci with the response to etanercept biosimilar. Regional association plot with the significance of the SNPs (i.e., -log10(P-value), y-axis) as a function of the basepair location on different chromosomes (x-axis). The variant most associated with the response to etanercept biosimilar is colored in purple and the remaining SNPs are colored according to the linkage disequilibrium (r^2^ value) with this SNP. The estimated recombination rates (centiMorgans/Megabase, right y-axis) are plotted as a continuous background line (blue).

Figure S4. The regional plot of rs11801616.

Figure S5. The regional plot of rs3754679.

Figure S6. The regional plot of rs2431355.

Figure S7. The regional plot of rs13166823.

Figure S8. The regional plot of rs10220768.

Figure S9. The regional plot of rs4796752.

Figure S10. The regional plot of rs13045590.

**Supplementary Table S1.**

Meta-analysis using fixed-effects model result of 7 SNPs in the patients of good responders and poor responders after 12 weeks treatment.

| SNP |  | Chr | Position | Allele | P-value | OR (95%CI) | P_heterogeneity |
| --- | --- | --- | --- | --- | --- | --- | --- |
| rs11801616 |  | 1 | 31311684 | G | 1.50E-03 | 0.30 (0.14-0.64) | 9.04E-01 |
| rs3754679 |  | 2 | 101599393 | G | 5.79E-02 | 0.57 (0.31-1.02 | 1.35E-01 |
| rs2431355 |  | 5 | 75934205 | T | 7.18E-02 | 1.72 (0.95-3.11) | 1.56E-01 |
| rs13166823 |  | 5 | 131752644 | G | 2.59E-02 | 0.41 (0.19-0.90) | 1.43E-01 |
| rs10220768 |  | 15 | 24319693 | C | 1.39E-05 | 3.72 (2.06-6.73) | 8.10E-01 |
| rs4796752 |  | 17 | 39629843 | C | 1.71E-05 | 3.63 (2.02-6.54) | 4.86E-01 |
| rs13045590 |  | 20 | 57575102 | T | 5.63E-04 | 0.20 (0.08-0.50) | 5.86E-01 |

Abbreviations: SNP, single nucleotide polymorphisms; Chr, chromosome OR, odds ratio; CI, confidence interval for odds ratio.

.

**Supplementary Table S2.**

The most significant eQTL analysis results of six SNPs

| SNP | chr | Gene Symbol | P-value | NES | Tissue |
| --- | --- | --- | --- | --- | --- |
| rs16834083* | 1 | *SDC3* | 6.40E-09 | -0.26 | Artery - Tibial |
| rs3754679 | 2 | *CNOT11* | 1.00E-06 | 0.16 | Breast - Mammary Tissue |
| rs2431355 | 5 | *F2RL2* | 3.70E-19 | -0.35 | Nerve - Tibial |
| rs7181324* | 15 | *NPAP1* | 7.8E-06 | -0.18 | Thyroid |
| rs4796752 | 17 | *KRT31* | 1.80E-11 | 0.25 | Skin - Sun Exposed (Lower leg) |
| rs13045590 | 20 | *CTSZ* | 9.50E-23 | 0.39 | Muscle - Skeletal |

Abbreviations: Chr, chromosome; SNP, single nucleotide polymorphisms; NES, normalized effect size.

*rs11801616 and rs10220768 are in high LD with rs11801616 and rs10220768 respectively.
